# XDeathDB: a visualization platform for cell death molecular interactions

**DOI:** 10.1038/s41419-021-04397-x

**Published:** 2021-12-14

**Authors:** Venkat Sundar Gadepalli, Hangil Kim, Yueze Liu, Tao Han, Lijun Cheng

**Affiliations:** 1 Research Information Technology, College of Medicine, Ohio State University, 1585 Neil Ave, Columbus, OH 43210 USA; 2 1Department of Biomedical Informatics, The Ohio State University, Columbus, OH 43210 USA; 3 The Grainger College of Engineering, The University of Illinois-Urbana-Champaign, Urbana and Champaign, Champaign, IL 61801 USA

**Keywords:** Cell division, Cancer

## Abstract

Lots of cell death initiator and effector molecules, signalling pathways and subcellular sites have been identified as key mediators in both cell death processes in cancer. The XDeathDB visualization platform provides a comprehensive cell death and their crosstalk resource for deciphering the signaling network organization of interactions among different cell death modes associated with 1461 cancer types and COVID-19, with an aim to understand the molecular mechanisms of physiological cell death in disease and facilitate systems-oriented novel drug discovery in inducing cell deaths properly. Apoptosis, autosis, efferocytosis, ferroptosis, immunogenic cell death, intrinsic apoptosis, lysosomal cell death, mitotic cell death, mitochondrial permeability transition, necroptosis, parthanatos, and pyroptosis related to 12 cell deaths and their crosstalk can be observed systematically by the platform. Big data for cell death gene-disease associations, gene-cell death pathway associations, pathway-cell death mode associations, and cell death-cell death associations is collected by literature review articles and public database from iRefIndex, STRING, BioGRID, Reactom, Pathway’s commons, DisGeNET, DrugBank, and Therapeutic Target Database (TTD). An interactive webtool, XDeathDB, is built by web applications with R-Shiny, JavaScript (JS) and Shiny Server Iso. With this platform, users can search specific interactions from vast interdependent networks that occur in the realm of cell death. A multilayer spectral graph clustering method that performs convex layer aggregation to identify crosstalk function among cell death modes for a specific cancer. 147 hallmark genes of cell death could be observed in detail in these networks. These potential druggable targets are displayed systematically and tailoring networks to visualize specified relations is available to fulfil user-specific needs. Users can access XDeathDB for free at https://pcm2019.shinyapps.io/XDeathDB/.

## Introduction

Cell death is an essential biological process for physiological growth and is used during developmental stages when tissues begin to form and shape [1]. Abnormalities of cell death programs contribute to several important diseases such as cancer, Alzheimer’s disease, autoimmune diseases, and chronic inflammation, where tissues or organs progressively deteriorate over time [2]. The three fundamental types of programmed cell death are apoptosis (type I cell death), autophagic (type II), and necrosis (type III) [1, 3]. Apoptosis is triggered when cell-surface death receptors, such as Fas is bound by their ligands (the extrinsic pathway) or when Bcl2-family pro-apoptotic proteins cause the permeabilization of the mitochondrial outer membrane (the intrinsic pathway) [4]. Autophagy is defined as a catabolic process in which parts of the cytosol and specific organelles are engulfed by a double-membrane structure, known as auto-phagosomes, and eventually degraded [5]. Necrotic cell death is characterized by the rapid loss of plasma membrane integrity. Recently, new cell death modes in cell biology, such as efferocytosis [6], mitochondria [7], and parthanatos [8] etc. have been recognized. A growing number of novel forms of cell death have been identified [9] as the so-called ‘Valley of Death’ [10]. The Nomenclature Committee on Cell Death (NCCC) [3, 11] classified twelve cell death modes based on biochemical and cellular characteristics, which are autophagy, autosis, necroptosis, efferocytosis, ferroptosis, immunogenic cell death, intrinsic apoptosis, lysozomal cell death, mitotic cell death, mitochondria permeability transition cell death, parthanatos, and pyroptosis.

For a long time, distinct modes of cell death were studied in isolation, so the prevailing cell death database suggested that they represented mutually exclusive cellular states, and none of them report the crosstalk (associations) of cell death modes. Current cell death databases often only outline molecular function in three basic modes of cell death [12]: apoptosis, autophagy, and necrosis. Ten databases related to human cell death have already been constructed. Database “Apoptoproteomics” [13], “Deathbase” [14], “ApoCanD” [15], “Degrabase” [16] present quantitative data of relevant pathway proteins for apoptosis cell death mode. “MEROPS” [17], “CASBAH” [18], “CASPDB” [19], “MerCASBA” [20] and “CaspNeuroD” [21] mostly focus on active caspases and their N terminus. “Autophagy Regulatory Network” explores only autophagic protein interactions while “BCL2DB” [22] presents BCL-2 family members and BH3 only proteins. “NcRDEATHDB” [23] presents non-coding RNA mediated cell death and lncRNA mediated cell death interaction with protein–protein interaction networks in apoptosis, autophagy, and necrosis as an updated version of ncRDeathDB. Until now, there is no database that comprehensively characterizes and annotates 12 cell death modes and their crosstalk.

Cell death displays distinct morphological features by activating signalling pathways. For example, the extrinsic and intrinsic cell death signalling pathways manifest apoptotic phenotypes facilitated by ‘effector’ caspases-3 and caspase-7, caspase-8 and caspase-9 [24]. The ‘extrinsic’ pathway transduces the signals of extracellular ‘death ligands’ belonging to the TNF superfamily (e.g., TNF-beta, Fas ligand [FasL)/Apo1L/CD95L, Trail/Apo2L, Apo3L) [25]. An accumulation of data testifies many deaths do not occur as an independent event but within the interactive machinery of intracellular or inter-cellular events [26], which requires various cascades of proteins/genes belonging to different cell death modes that participate in crosstalk [27, 28]. Suppression or activation of one type of cell death, such as autophagy, can influence the activity of another type, like apoptosis and necroptosis [29]. On the other hand, an accumulation of data testifies the existence of cross talk between cell death pathways at the molecular level are related to pathogenesis not only cancer, but also other diseases, such as COVID-19, HIV, inflammation, and cardiovascular disease [30, 31, 36]. Commonly seen in hepatocellular carcinoma, both necroptosis and intrinsic apoptosis occur due to hypoxia or toxic injury, and both are activated through the TNF-induced cell death pathway. In cardiovascular physiology, autophagy and mitochondrial transition permeability cell death can both be inhibited by Fbxo32, Lamp2, miR-212, Pink1, Atg5, and Trp53 [30]. Thousands of papers have been published on these topics [28, 31], for example, between the crosstalk of autophagy and apoptosis [28], pyroptosis and necroptosis [32, 33], and mitochondria activating caspase proteases through the intrinsic pathway of apoptosis [27]. Surprisingly, there is no database that carefully and explicitly documents crosstalk between specific pairs of signalling pathways of cell death modes and key genes. Identified shared genes and pathways can be candidate targets for novel drug treatments for cell specific effects in cancer and COVID-19 [33, 34]. Therefore, a cell death repository clarifying shared genes and signalling pathways among cell death modes is urgently necessary.

To interpret the molecular mechanism of cell death modes comprehensively and their crosstalk knowledge and support medical research, genome analysis, systems biology, and education, we developed XDeathDB, an intuitive bioinformatics tool as an R Shiny application to understand the pathogenic mechanisms related to cell death. XDeathDB characterizes cell death signalling pathways in different cell death types upon diverse death triggers. These hallmark genes and signalling pathways and drug targets are systematically annotated related to various cell death modes. XDeathDB recognized that cell death can occur by genetically controlled processes and has enabled abilities to unravel the mechanisms of cancer. By the platform, users can explore crosstalk of cell death modes on genes and pathways within a simple interface. As a result, it will improve the knowledge behind the initiation of cell death programs and the relevant signalling pathways, which would facilitate the development of pharmacologic agents that initiate or inhibit programmed cell death properly. Searching molecular mechanisms in gene-cell death, pathway-cell death, cell death-cell death, cell death-disease association and identifying key genes and the cell death-based treatment in each cancer type, are goal of XDeathDB. (1) For example, cells’ resistance or survival from one type of cell death, such as apoptosis [35, 36], can modify their response to damage and eventually activate an alternative cell death mode such as ferroptosis by dialogue between different pathways. (2) Cell death is triggered by key proteins which induce growth or suppression, such as tumour oncology genes, and therefore, have become important drug targets for cancer therapies [37]. By the platform, user can search the cell death-based treatment in cancer as well [36]. (3) In XDeathDB, we can search and observe these hallmark genes of cell death among crosstalk directly and understand the mechanism of disease or resistance, for example, protein Bid has been recognized as a key molecular switch between intrinsic and extrinsic apoptotic pathways during disease progression [38].

## Methods

### XDeathDB repository

**XDeathDB** cell death knowledge base contained human 27,654 genes, 149 cell death hallmark(key) genes, 1235 drug target genes, 2213 drugs, 164 signalling pathways, 12 cell death modes and 1461 cancer types and COVID-19. All associations include 461,711 gene-disease associations (GDAs) between 12,466 genes and 1461 cancer types and COVID-19, 596,521 gene-cell death pathway associations between 2275 genes and 164 cell death pathways and cell death-cell death associations with 21,488 unique gene-gene interactions by literature review articles and public database from DisGeNET [39], iRefIndex [40], STRING [41], BioGRID [42], Reactom [43], Pathway’s commons [44], DrugBank [45], and Therapeutic Target Database (TTD) [46]. A breakdown of the data collected of each cell death mode is summarized in Table 1. All these entity relationships are summarized in Table 2. These molecular interactions cover signal transduction, protein–protein interactions, activation, membership, phosphorylation, regulation of binding, molecular cleave, localization, modification, genetic variation, and drug-targets inhibition. (See Table 2. entity relationships).

**Table 1:** Data collection of key proteins (key genes), pathways, cancer types, cell death modes and their associations in XDeathDB database.

Cell death modes	#Genes	#Key-genes	#Path-ways	Network analysis		
				#Sub-networks	#Genes	#Pairs of genes
Apoptosis	395	18	10	12	291	740
Autophagy	368	8	16	8	245	2977
Autosis	2093	2	58	25	876	2944
Efferocytosis	30	9	1	6	211	2112
Ferroptosis	495	1	2	16	560	2410
Immunogenic cell death	3917	9	12	25	877	2307
Intrinsic apoptosis cell death	59	20	3	8	281	2188
Lysozomal cell death	339	8	8	8	247	1175
Mitotic cell death	3627	21	8	24	36	321
Mitochondrial permeability transition (MTP)	306	10	2	8	55	252
Necroptosis	411	5	7	8	176	1913
Parthanatos	473	22	10	10	351	2523
Pyroptosis	852	3	16	13	281	1863
Proliferation	1495	12	11	11		
1461 cancer types	12,466		164			
COVID-19	1832					

Sub-networks is the number of networks within one cell death mode. Pairs of proteins is the number of interactions or “edges” and proteins are “nodes”.

**Table 2:** Entity relationships in XDeathDB knowledge base.

Relationship Labels	Entity relationships	Number of genes	Number of edges
PP	Protein–protein binding	2039	5129
A	Activation	1090	3980
E	Expression(includes metabolism/synthesis)	1286	3469
RB	Regulation of binding	709	1739
P	Phosphorylation	570	1531
LO	Localization	645	1437
M	Membership	734	743
MC	Molecular cleavage	383	707
T	Translocation	272	691
I	Inhibition	357	608
T	Transcription	277	465
MO	Modification	174	332
PD	Protein–DNA interactions	239	318
U	Ubiquitination	181	266
PR	Protein–RNA interactions	50	48
CP	Chemical-protein interactions	1251	2010

**Fig. 1:**
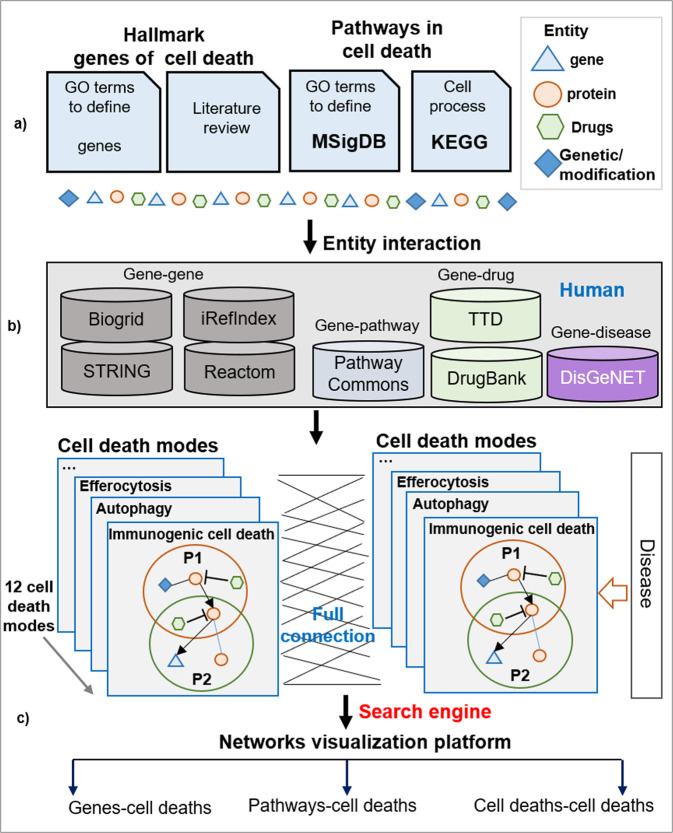
Framework of XDeathDB. **a** Collection of developmental pathways and hallmark genes of cell death by 72 literature reviews. **b** Entity interaction data collected from public data base. **c** Building R-Shiny interactive webtool for cell death function and crosstalk searching while visualization.

An interactive webtool, XDeathDB, is built by web applications with R-Shiny, JavaScript (JS) and Shiny Server Iso. XDeathDB is organized by analyzing shared gene targets or pathways and the responses between two cell death modes either in the scope of disease molecular mechanisms or in regular biochemical processes. It incorporates genes, pathways, and interaction networks data to outline twelve different cell death modes. All data is curated from both multiple public databases and literature review by the sequential steps (**a**) and (**b**). Then a visualized interaction platform is developed in step (**c**) (Fig. 1).

### Twelve cell death modes

**XDeathDB** includes intrinsic apoptosis, extrinsic apoptosis, mitochondrial permeability transition (MPT)-driven necrosis, necroptosis, ferroptosis, pyroptosis, parthanatos, entotic cell death, NETotic cell death, lysosome-dependent cell death, autophagy-dependent cell death, immunogenic cell death, cellular senescence, and mitotic catastrophe by Nomenclature Committee on Cell Death (NCCD) provides molecularly oriented definitions of 12 cell death modes in year 2018 [3].

### Hallmark genes of cell death

Seventy-two literature reviews of the cell death recommendations by the Nomenclature Committee on Cell Death 2018 [1, 3] was used to draft a preliminary inventory of fundamental key genes that characterized cell death modes and to collect data regarding function and relation for that specific cell death modes [51]. In cell death mechanisms, a specific gene was identified from literature that defined its mechanism of action and responsibility. This gene is either a part of a cascade, which brings upon eventual activation or is the activator itself. This data set is continually expanding to bring about more hallmark genes by their relationships. One hundred and forty-nine hallmark genes for cell death were obtained. These hallmark genes were qualifiable entities of crosstalk that could help explain the delicate balance between multiple cell death modes in various cancers.

### Cell death associated pathways

Cell death associated pathways by the 12 cell death modes and the 149 hallmark genes are referred to as terms and all terms are mapped to Gene Ontology (GO) [47], Kyoto Encyclopaedia of Genes and Genomes (KEGG) [48], and Molecular Signatures Database (MSigDB) [49] v6.2 C5 GO (annotated pathway gene sets) to seek pathways sets associated with cell death. A total of 164 pathways related to 2,275 genes were obtained. In which, 7 pathways are from KEGG, hsa04140, Autophagy; hsa04110, Cell cycle; hsa04210, hsa04215, Apoptosis; hsa04216, Ferroptosis; hsa04217, Necroptosis; hsa04115, Apoptosis-p53 signalling pathway. Because immune related cell death is complex, we only covered 8 cell death pathways: NToll-like receptor signalling pathway [50, 51] NT cell receptor signalling pathway, T cell receptor signalling pathway, B cell receptor signalling pathway, MARK pathway, TGF_BETA signalling pathway and NOTCH signalling pathway.

### XDeathDB entities annotation

Gene annotation is by HUGO Gene Nomenclature Committee HGNC (https://www.genenames.org) and drug annotation is based on DrugBank [45].

#### Entity interaction

Through a second round of data collection, interactions and networks of cell death were incorporated. These 164 pathways related to 2275 genes are mapping to each public database to seek the gene-gene causality interaction. Figure 1 describes the automated construction of the cell death interaction database by searching curated protein–protein interaction (PPI) data, genetic mutation, transcriptome data and protein modification from public database iRefIndex, STRING, BioGRID, Reactom, Pathways commons, DrugBank, TTD. All these gene-gene interaction integrated with gene-disease interaction dataset from DisGeNET database. Integrating 21,488 unique gene-gene associations and 461,711 gene-disease associations (GDAs) data and setting up comprehensive entity interaction table.

### Original data source of interactions

**iRefIndex** [40] (version 17, https://irefindex.vib.be/wiki/index.php/README_MITAB2.6_for_iRefIndex_17.0)^.^ RefIndex provides an index of protein interactions available in a number of primary interaction databases including BIND, BioGRID, CORUM, DIP, HPRD, InnateDB, IntAct, MatrixDB, MINT, MPact, MPIDB, MPPI, Reactome, VirHostnet, and QuickGO.

**STRING** [41] (https://string-db.org/), provides Protein–Protein Interaction (PPI) networks in Humans.

**BioGRID** [42] (version 4.1.190, https://thebiogrid.org/) provides 73,317 publications for 1,928,373 protein and genetic interactions, 28,379 chemical associations and 874,796 post translational modifications from major model organism species. Only human’s protein and genetic interaction data is extracted.

**Reactom** [43] Pathway Database (version 46, http://www.reactome.org) is a curated, peer-reviewed resource for human biological processes, including the pathways of intermediary metabolism, regulatory pathways, and signal transduction, and high-level processes, such as the cell cycle. The current version 46 provides 7088 human proteins (34% of the predicted human proteome), participating in 6744 reactions based on data extracted from 15,107 research publications with PubMed links.

**Pathway’s commons** [44] (version 12, https://www.pathwaycommons.org/) contains data from 22 pathway and interactions databases. It provides 5772 detailed human biochemical processes (i.e. pathways) and ∼2.3 million interactions in biological pathways including biochemical reactions, assembly of biomolecular complexes, transport and catalysis events and physical interactions involving proteins, DNA, RNA, and small molecules (e.g. metabolites and drug compounds). Only human’s data is extracted here.

**Drug-target interactions** were obtained from database **DrugBank** [45] and **Therapeutic Target Database** [46] (http://db.idrblab.net/ttd/).

**DisGeNET** [39] (version 7.0, https://www.disgenet.org/) collected gene-disease associations (GDAs) and variants associated to human diseases by text mining MEDLINE abstracts using the BeFree system. We extracted 461711 GDAs between 12466 genes and 1461 cancer types and 1832 genes associated with severe acute respiratory syndrome coronavirus 2 (COVID-9) from DisGeNET and kept its original four scores------ Disease Specificity Index (DSI), Disease Pleiotropy Index (DPI), Evidence Index (EI) and Score, the detailed refers to literature [39, 46].

#### Gene Ontology biology processing (pathway) networks

The Gene Ontology (GO) knowledgebase [47] (http://geneontology.org/) is the world’s largest curated database of previously published findings on the functions of genes. MSigDB Molecular Signatures Database (2020, V7.4) [42] C5 organized ontology gene sets for performing gene set enrichment analysis. To date, we have organized 9996 GO biology process (pathway) with 21,000 genes from GO and MSigDB. Mapping all these gene sets to database Pathway Commons to obtain the Gene Ontology biology processing (pathway) networks (Fig. 1).

#### Molecular function annotation to each of cell death

To each of cell death mode, we did an annotation for molecular function based on pathway. (1) Genes in each of cell death mode were overlaid onto the Gene Ontology biology processing networks Fig. 1(i) - (ii). Focus genes were identified as the subset having direct interaction(s) with other genes in the database. While subnetworks consist of a relatively small number of deeply characterized regulatory and signalling events represented as directed graphs, each cell death network is separated as 25–30 gene-sets within subnetworks. Specific cell death molecular functions occur, are presented by the subnetwork relationships/regulations variations. Subnetwork modules and associated molecular function for 12 cell death modes were identified and annotated systematically.

**Fig. 2:**
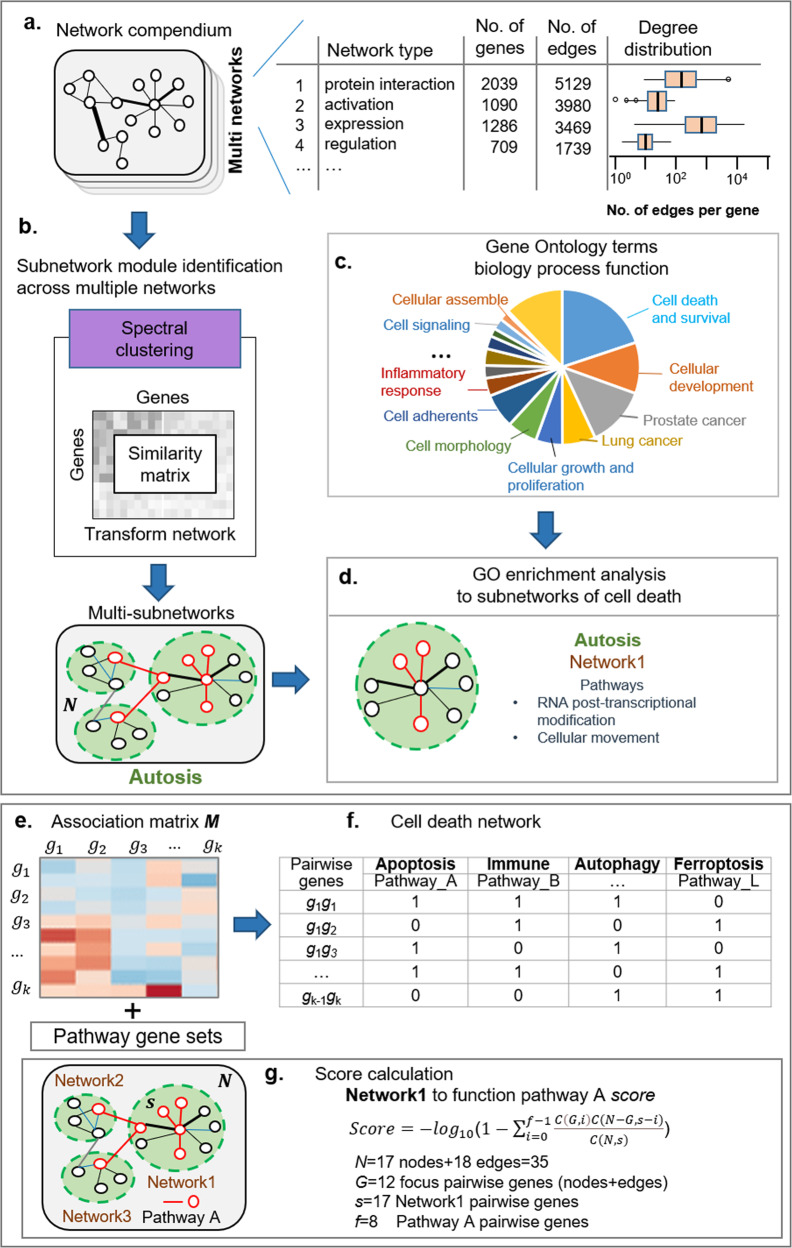
Molecular function annotation to a specific cell death by network module analysis. **a** Network types included in the knowledgebase. The boxplot centre lines show the median, box limits show upper and lower quartiles, whiskers show 1.5× interquartile range and points show outliers. **b** Spectral clustering is for module identification approach. **c** Outline of the subnetwork function annotation by GO. **d** Subnetworks of cell death enrichment function analysis. A hypothetical example of detecting cell-death mode-mode functional relationships by score statistics. **e** In the weight score matrix *M* between gene *i* and gene *j*. Here, the node is on the diagonal elements where *i* = *j* in the array; the edge is on the elements where *i≠j* in the array. **f** All redundant and structured terms are broken into “independent” terms, such as cell-death modes. Association of each node (gene) and edge (pair of genes) with some of the annotation-term collection allows the construction of a gene-gene annotation matrix in a binary format, where one represents a positive match for the gene term and zero represents the unknown. Thus, each cell-death mode-mode annotation has a unique profile of terms represented by a combination of ones and zeros. **g** The significant biology process score calculation to each subnetwork.

Molecular function annotation to each of cell death mode (called network analyses) is a series of four “steps” that we follow in a set order (Fig. 2).

(**a**) The XDeathDB knowledgebase collected a novel role for cell death signalling pathways on different levels, including gene regulations, protein interactions on DNA, RNA and proteins, translocation, membership, molecular cleavage, and post-translational modifications, such as phosphorylation and ubiquitination, etc. in the intracellular signalling network. A total of sixteen network types are included (Table 2). Then, all the networks are integrated together. A gene degree is the number of edges of connection to the gene. This different mechanism often shows a high degree of association between the different gene sets. Degree distribution to each of specific network was observed. High connection degrees are in regard to key genes in following clustering analyses.

(**b**) Many bioinformatics methods [52] have been proposed for reducing the complexity of large gene or protein networks into relevant subnetworks or modules. The latest open competition [52] to comprehensively assess network module identification methods across diverse networks showed the best performing method is spectral clustering [53], which recovers complementary trait associated modules in the DREAM challenge [52]. Here, the compact integration of multi-network topology and functional analysis of genes refers to literature [54]. The spectral clustering method [52] is used to identify the main types of subnetwork module in the integrated network. Each cell death network is separated as 25–30 gene-sets subnetworks to each cell death fusion network. These focus genes of highly interconnected genes in the Gene Ontology biology processing network were identified by statistical likelihood using the following equation refers to literature [55]

1 $${\rm{Score}} = - {\log}_{10} \left(1 - {\sum \limits_{i = 0}^{f - 1}}\frac{{C\left( {G,i} \right)C(N - G,s - i)}}{{C(N,s)}} \right)$$

where *N* is the number of pairwise genes in the Gene Ontology biology processing network, of which *G* are focus pairwise genes, for a pathway of *s* pairwise genes, *f* of which are focus pairwise genes, *C*(*n,k*) is the binomial coefficient. Pathways with a score >4 (*P*_value < 0.0001) were combined to form a composite network representing the underlying biology of the process. The calculation of scores used to rank subnetworks of cell death (Fig. 2e–g). The top 25 subnetworks and associated molecular function of each cell death network are picked up for visualization.

(**c**) Network function knowledge base. Pathway Commons [44] networks capture genome-wide interactions derived from integrating 20 interaction databases, which are represented as binary, undirected graphs, capture direct, indirect, and discovered regulatory interactions and can be understood to capture complex cellular logic as simplified connections between pairs of genes. Thus, Pathway Commons networks provide more flexibility for the discovery of novel biological mechanisms underlying disease phenotypes. MSigDB [49] C5-gene ontology gene sets collected the Gene Ontology resource (GO) which contains Biology Progress (BP), Cell Components (CC), and Main Function (MF) components and the Human Phenotype Ontology (HPO). Mapping MSigDB [49] C5-gene ontology BP gene sets (14,765 genes) to Pathway Commons networks is our basic interaction knowledge platform.

(**d**) Outline of the subnetwork function annotation. The integrating networks are like pathways in that both consist of interacting biomolecular entities affecting specific cellular functions. The system implements Fisher’s exact test to determine whether a subnetwork is enriched with genes and edges of each signalling pathways in the above network function knowledge base.

A total of 122 subnetworks are identified among 12 cell death modes. Specific to each of cell death (Table 1), all subnetworks’ functions are annotated and stored in XDeathDB for searching.

### XDeathDB platform

**Fig. 3:**
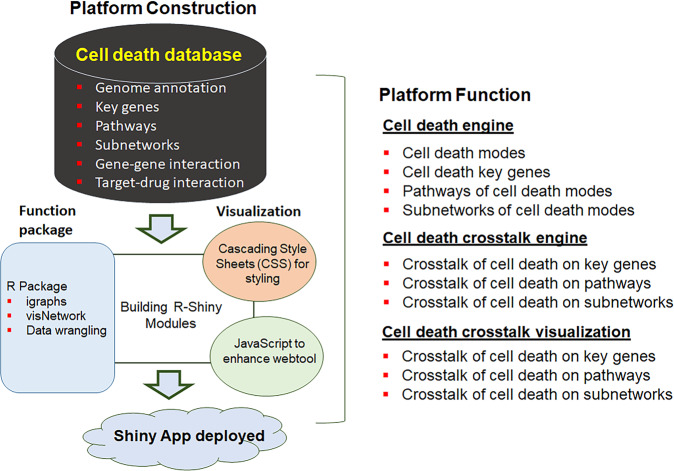
XDeathDB website platform construction framework and functions. The XDeathDB platform (on the left) data was shared through a client-server architecture with a web user interface (UI), front-end interface, and backend server components. R, Python, R-Shiny, HyperText Markup Language (HTML), javaScript and Structured Query Language (SQL) are the main programme languages for the tool development. XDeathDB is deployed with the advanced cloud R Shiny application (shinyapps.iso). Web user-interface (UI) development: Python, R, Hypertext Markup Language (HTML), customized Cascading Style Sheets (CSS), JavaScript programming languages, and Structured Query Language (SQL) are integrated under the R-Shiny application framework during development. The R-Shiny App application server provides access to the data and visualization for the clients on different devices since it is not platform specific. Front-end interface: R packages R (>3.3.0), including igraphs, visNetwor, and data wrangling etc. are used for network dynamic visualization. Shiny (R package), Opencpu (R package), reticulate (R package, providing an R interface to Python modules, enabling seamless, high-performance interoperability), cloud database and a web browser. Backend server side: All cell death database and management services are built in the structured query language (SQL) database server SQLite with the relational database management system (RDBMS). Four tables relating to drugs, genes, variants, and diseases are designed individually in SQLite server. The XDeathDB functions (on the right) include (1) a comprehensive search engine of each mode of cell death and their interaction at the gene and pathway relevant to the regulation of cell death. (2) XDeathDB has implemented crosstalk enrichment analysis on cell death at gene, pathway, and cell death mode levels for each cancer type. This allows users to freely select and combine these subnetwork modules to obtain their own network construction. (3) The visualization of crosstalk subnetworks among gene–cell death, pathway–cell death, and cell death–cell death for each cancer type. Users can identify the key target candidate and associated molecular function by the visualization observation.

The XDeathDB knowledgebase (https://pcm2019.shinyapps.io/XDeathDB/) is a comprehensive network map for cell death in a single consistent data visualization framework. The XDeathDB application platform is deployed with the advanced cloud Shiny app (shinyapps.iso) [56], which is offered as a self-service platform for shiny users to easily share shiny apps (Fig. 3). Integrating the R package, JavaScript, Shiny Server Iso, XDeathDB creates clear interactive visual applications through the search engine for cell death modes. This involved building components of the web user-interface (UI) and the server components that can be composable to build the app tool. Various R packages were employed, including tidyverse [57], dplyr [58], visNetwork [59], igraph [60] and networkD3 (D3 JavaScript Network Graphs from R, https://d3js.org/) to perform data wrangling, data transformation and generating interactive network plots by using Hypertext Markup Language (HTML) or Scalable Vector Graphics (SVG). Further enhancements of the app were done by custom Cascading Style Sheets (CSS) for styling and the functionality of the apps are enhanced by custom JavaScript (JS) codes. JavaScript programming aggregated interaction networks on each of signal transduction, transport, DNA replication, metabolism, post translational modifications of other cellular processes and protein interaction event. 16 relationships as an ordered network of molecular transformations. At the same time, multiple sub-networks could be integrated easily depending on the user’s requirements.

### XDeathDB function

The goal of the current web tool is to provide a one-stop source for researchers to mine through the different genes, pathways, drug targets involved in specific cell death modes as well as their crosstalk.

#### Cell death search engine

1. Comprehensive search functions of cell death for genes
  Cell death modes’ defining boundaries become elusive after considering specific extra- and intra-cellular conditions. Therefore, maintaining those boundaries starts with identifying which gene or protein is responsible for the mediation between the two cell modes or multiple cell death modes. Genes can be visually located in interactive networks and user-decided interactions.
  The search function in the “**Gene Network**” tab will pull up any data matching the search input as the input is valid for genes, locations, family, and drugs. The network data provides information of its function in which its respective cell death mode occurs, and the relationships, regulations, and genes associated with it.
  Users can input a desired gene to locate the gene in multiple cell death modes, which provides their first insight to where possible crosstalk occurs between cell deaths modes. We emphasized the key genes during cell death, determined by literature that clearly indicates its considerable influence. Searching capabilities allow users to search key genes throughout the entire database, not just within one cell death mode therefore the filters they choose allow them to assemble lists of key genes of any combination of cell death modes, pathway involvement shown in Fig. 4A under the “**Cell Death Engine**” tab in “Key genes annotations”. The lists show location, entrez gene name, synonyms, and symbol, with a direct link to relevant validated publication, and in what other cell death mode it is a key gene.

**Fig. 4:**
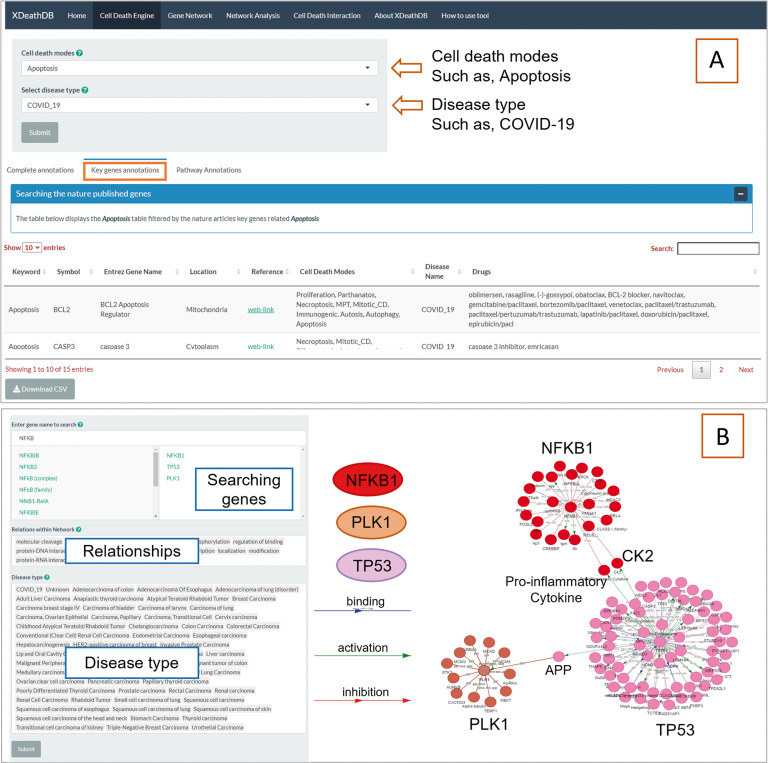
Key genes and network searching for cell death. **A** Cell death key genes; **B** Cell death crosstalk networks by user input gene sets.
  All the while, users can also build their own gene networks, drill down their own data, search across the database, and download the respective plots and data. The present genes in the network that are responsible for observing the cell death modes’ switching can serve as the in-between mediator from some biochemical action leading to cell death. This provides information on possible drug targets and therapies. For example (Fig. 4B), we searched for crosstalk between autophagy and immunogenic cell death. Immunogenic cytokine NF-κB is increasingly recognized as a crucial player in many steps of cancer initiation and progression [61]. NF-κB represents a central factor in inflammation or proliferation as well as cell death by crosstalk PLK1 and TP53 in proinflammatory cytokines. NF-κB is known as an important therapeutic target. When NF-κB inhibited by a genetic or pharmacological molecule, autophagy is induced, as seen in ovarian cancers. However, through the autophagic PLK1 induction of cell death, the NfKB1-TP53 pathways induce immunogenic-mediated cell death. The phenomenon is disclosed in crosstalk of cell death modes (Fig. 5) as well.
2. Comprehensive searching for molecular functions of cell death in subnetworks

**Fig. 5:**
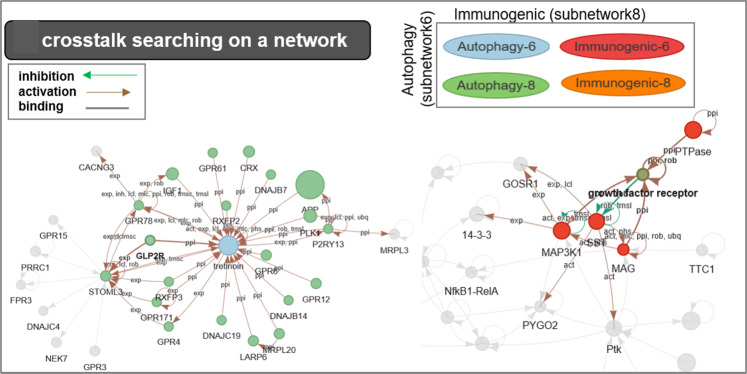
A pathway’s crosstalk of cell death modes in XDeathDB. As crosstalk of immunogenic cell death and autophagy cell death for an example, we observed the crosstalk of T cell receptor signaling pathway (immunogenic subnetwork 8) and the autophagy subnetwork 6 under a network here. Key druggable target genes are annotated.

**Fig. 6:**
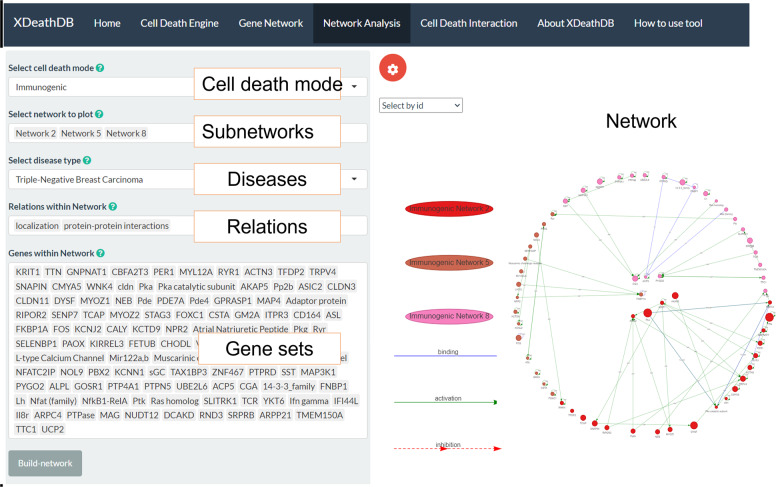
Sub-networks to a specific cell death and associated molecular function annotation. Cell death modes, subnetwork enriched molecular function, cancer type, involved relations, and genes within networks are shown on the right panel. The associated network visualization on the left panel is provided too.

The molecular function analyses intend on connecting cell death processes that are embedded within mechanisms geared to maintain overall stability. In “**Network Analyses**,” networks can be interactively visualized and distinguish specific genes as shown in Fig. 6. The purpose of this function is to allow users to see direct relationships of a gene within an entire sub-network. Users can choose any network of any cell death mode to visualize their crosstalk along with quick references to their actual function, which allows the user to try a large number of combinations of networks and pathways. In addition, genes can be filtered to be emphasized in a network. To expand on the data above, crosstalk was explored between the data above and function analyses in Table 1.

#### Cell death crosstalk engine

The subnetworks are the main highways of information exchange in the biological process. Because networks are organized under respective cell death modes, they automatically provide a molecular setting in which they operate. In this process, the phenomenon called crosstalk will be discovered between the subnetworks. The crosstalk of cell death modes in subnetworks can be manually searched from our knowledge base. The visualization of molecular subnetworks interactions annotated in web resources is useful to offer users such information in a clear intuitive layout. These interactions are frequently represented as crosstalk events that are laid out in free network space where different cell death modes and functions (subnetworks) are individually recognized (Fig. 5).

Users can search any network of any cell death mode to visualize them along with quick references to the actual function on subnetworks. In addition, users can filter or search specific genes to design a network that emphasizes genes. These subnetworks can impart insight on the changes of conformation within proteins and biological pathways. Because these networks work under the constraints of certain conditions of a disease or pathological state, they provide a framework of how they are utilized. The breakdown of pathways’ interaction shows critical points where crosstalk occurs.

## Discussion

Common pattern we observed is that cell death modes often work in conjunction to another, not as exclusive events. Therefore, the ability to search any function, gene, or pathway in respect to a cell death mode offers a new level of discussion in regard to the balance of cell death depending on the status of the entire system, a balance that is determined by the pathways of interaction and crosstalk with other cell death modes. XDeathDB is useful and extensively comprehensive to serve as a “headquarters” for cell death research regarding crosstalk and molecular function by unique subnetwork.

XDeathDB is a free open source for data source, JavaScript and R-Shiny library, which offers a series of pre-defined elements and interaction types meant to facilitate the representation of cell death modes subnetworks consisting of causal interactions without neglecting simple protein–protein interaction and gene regulation networks etc. Twelve cell death modes have been identified with key genes, pathways, network, and crosstalk literature data. Through the platform, users can perform gene searches and find a relevant or shared gene within all cell death modes so novel relationships will emerge through the identification of common genes between cell death modes and therefore processes that were thought to have occurred independently. Evaluating the ubiquitous nature of a certain gene stipulates the importance of its role or its diverse capabilities. Specifically, we show that determining key genes allows for the creation of new, advanced drug treatments, which can efficiently target problematic genes within multiple cell death pathways. The focus of crosstalk is shifting to the crosstalk events that occur to expanding drug therapies for diseases from cancer to autoimmune maladies.

Its network analyses can visualize multiple networks from multiple different cell death modes to show all of the possible interactions that could occur. Through visualization and integration, the database caters to user-specific needs to tailor network and crosstalk information. Therefore, the platform, with its dense user-configured output capabilities, can build countless interaction map. This allows for more than 1000 different combinations of networks analyses to be visualized all with tailored gene annotations and regulation types.

XDeathDB provides the community with access to data regarding the implications of cell death modes’ authority in the human system and ways to possibly manipulate them for future advancements. We ultimately hope this database develops a deeper understanding of how cell death modes can be manipulated for drug advancement and future therapies for diseases such as COVID-19 and cancers. In the future, XDeathDB functions both as an archive of biological processes and multiple interaction network as a tool for discovering unexpected cell death functional relationships in data such as gene expression profiles or somatic mutation catalogues from tumour cells.

## Data Availability

Gene interaction network data that support the findings of this study have been deposited in the XDeathDB platform, which is available at https://pcm2019.shinyapps.io/XDeathDB/.
